# Granulocyte-colony stimulating factor (G-CSF) and granulocyte-macrophage colony stimulating factor (GM-CSF) for sepsis: a meta-analysis

**DOI:** 10.1186/cc10031

**Published:** 2011-02-10

**Authors:** Lulong Bo, Fei Wang, Jiali Zhu, Jinbao Li, Xiaoming Deng

**Affiliations:** 1 Department of Anesthesiology, Changhai Hospital, Second Military Medical University, 168 Changhai Road, Shanghai, 200433, PR China

## Abstract

**Introduction:**

To investigate the effects of G-CSF or GM-CSF therapy in non-neutropenic patients with sepsis.

**Methods:**

A systematic literature search of Medline, Embase and Cochrane Central Register of Controlled Trials was conducted using specific search terms. A manual review of references was also performed. Eligible studies were randomized control trials (RCTs) that compared granulocyte-colony stimulating factor (G-CSF) or granulocyte-macrophage colony stimulating factor (GM-CSF) therapy with placebo for the treatment of sepsis in adults. Main outcome measures were all-cause mortality at 14 days and 28 days after initiation of G-CSF or GM-CSF therapy, in-hospital mortality, reversal rate from infection, and adverse events.

**Results:**

Twelve RCTs with 2,380 patients were identified. In regard to 14-day mortality, a total of 9 death events occurred among 71 patients (12.7%) in the treatment group compared with 13 events among 67 patients (19.4%) in the placebo groups. Meta-analysis showed there was no significant difference in 28-day mortality when G-CSF or GM-CSF were compared with placebo (relative risks (RR) = 0.93, 95% confidence interval (CI): 0.79 to 1.11, *P *= 0.44; *P *for heterogeneity = 0.31, I^2 ^= 15%). Compared with placebo, G-CSF or GM-CSF therapy did not significantly reduce in-hospital mortality (RR = 0.97, 95% CI: 0.69 to 1.36, *P *= 0.86; *P *for heterogeneity = 0.80, I^2 ^= 0%). However, G-CSF or GM-CSF therapy significantly increased the reversal rate from infection (RR = 1.34, 95% CI: 1.11 to 1.62, *P *= 0.002; *P *for heterogeneity = 0.47, I^2 ^= 0%). No significant difference was observed in adverse events between groups (RR = 0.93, 95% CI: 0.70 to 1.23, *P *= 0.62; *P *for heterogeneity = 0.03, I^2 ^= 58%). Sensitivity analysis by excluding one trial did not significantly change the results of adverse events (RR = 1.05, 95% CI: 0.84 to 1.32, *P *= 0.44; *P *for heterogeneity = 0.17, I^2 ^= 36%).

**Conclusions:**

There is no current evidence supporting the routine use of G-CSF or GM-CSF in patients with sepsis. Large prospective multicenter clinical trials investigating monocytic HLA-DR (mHLA-DR)-guided G-CSF or GM-CSF therapy in patients with sepsis-associated immunosuppression are warranted.

## Introduction

Despite improvements in antimicrobial therapy and supportive care, the incidence of sepsis continues to rise and sepsis is now the third leading cause of infectious deaths in the United States [1], with a mortality rate ranging from 20% for sepsis to 50% for septic shock [2,3]. During the past decades, many clinical trials testing anti-inflammatory therapies have been performed. However, the effects of these approaches on patient mortality were rather disappointing [4-7]. It is now generally agreed that patients with sepsis are more prone to die in a state of sepsis-induced immunosuppression, including reduced monocytic phagocytotic activity, changes in monocytic cytokine expression, diminished monocytic antigen presentation, lymphocytic dysfunction and apoptosis-induced loss of circulating T-and B-lymphocytes [4,8-21]. Consequently, immunostimulatory therapies constitute an innovative strategy that deserves to be assessed for the treatment of sepsis [14,22,23].

To date, one approach is the use of granulocyte colony stimulating factor (G-CSF) or granulocyte-macrophage colony stimulating factor (GM-CSF), to augment myeloid cell functions in patients with sepsis. G-CSF, widely used in reducing the duration of febrile neutropenia following cytotoxic chemotherapy, has been shown to stimulate the production of neutrophils and modulate the function and activity of developing and mature neutrophils [24]. Compared to G-CSF, GM-CSF exhibits broader effects and induces proliferation and differentiation of neutrophils, monocytes, macrophages, and myeloid-derived dendritic cells. GM-CSF has been demonstrated to increase monocytic HLA-DR (mHLA-DR) expression and endotoxin-induced proinflammatory cytokine production in *ex vivo *whole blood cultures of patients with severe sepsis [25,26].

So far, G-CSF and GM-CSF have shown promise in the treatment of infection in non-neutropenic hosts in many animal models [27-30]. Additionally, several clinical trials have been conducted to investigate the effect of G-CSF or GM-CSF treatment in neonates and adults with infection. Recently, a meta-analysis investigating the effect of G-CSF and GM-CSF for treating neonatal infection showed no significant reduction in 14-day mortality [31]. To our best knowledge, no previous systematic review had been conducted to define the efficacy and safety of G-CSF and GM-CSF in patients with sepsis. Therefore, we attempted to summarize the available randomized control trials (RCTs) to determine whether G-CSF or GM-CSF therapy significantly reduced all-cause mortality at 14 days and 28 days, in-hospital mortality and occurrence of adverse events, and increased reversal rate from infection in patients with sepsis.

## Materials and methods

We followed the guidelines of Preferred Reporting Items for Systematic Reviews and Meta-Analyses for reporting our meta-analysis and results [32].

### Search strategy

We searched Pubmed, Embase, and the Cochrane Central Register of Controlled Trials (to 25 October 2010) to identify potentially relevant trials. We restricted the search to trials on adults and used the search terms "granulocyte colony-stimulating factor", "granulocyte colony stimulating factor, recombinant", "GCSF", "filgrastim", "lenograstim", "sargramostim", "pegfilgrastim", "granulocyte-macrophage colony-stimulating factor", "granulocyte-macrophage colony stimulating factor, recombinant", "GMCSF", "molgramostim", AND "sepsis", "septicemia", "septicaemia", "septic shock". We restricted the findings of this search with a highly sensitive search strategy recommended by the Cochrane Collaboration for identifying all randomized controlled trials [33]. In addition, we checked the reference lists of identified trials and previous relevant meta-analyses identified by the electronic search to find other potentially eligible trials. There was no language restriction for the search. Authors of papers were contacted when results were unclear or when relevant data were not reported.

### Study selection

We considered trials that investigated the therapeutic effects of G-CSF or GM-CSF administered intravenously or subcutaneously in adults with sepsis. Sepsis was defined according to the American College of Chest Physicians/Society of Critical Care Medicine consensus criteria [34] or was extrapolated to these criteria if not provided. Trials that allowed concurrent use of other therapies, including antibiotics, mechanical ventilation, steroids, bronchodilators and so on were included if they allowed equal access to such medications for patients in both arms of the trial. Randomized controlled trials specifically involving neutropenic patients or patients following chemotherapy were excluded. Agreement between reviewers regarding trial inclusion was assessed using the Cohen К statistic [35].

### Data extraction

Full text versions of all eligible trials were obtained for quality assessment and data extraction independently by two reviewers. Extracted data were entered into Microsoft Excel 2007 and were checked by a third reviewer. Disagreement or doubt was resolved by discussion. Abstracted data included study design (for example, date of conduct and sample size), patient characteristics, study methodology (for example, eligibility criteria, method of randomization, and blinding), intervention (for example, G-CSF and GM-CSF dosage, duration and route of administration) and main outcomes. The quality of trials was assessed with the methods recommended by the Cochrane Collaboration for assessing risk of bias [36]. The criteria used for quality assessment were sequence generation of allocation, allocation concealment, blinding, selective outcome reporting and other sources of bias. Each criterion was categorized as 'yes', 'no', or 'unclear', and the summary assessments of the risk of bias for each important outcome within and across studies was categorized as 'low risk of bias', 'unclear risk of bias' and 'high risk of bias'.

The primary outcomes of this meta-analysis were all-cause mortality at 14 days and 28 days after initiation of G-CSF or GM-CSF therapy. Secondary outcomes included in-hospital mortality, reversal rate from infection, and adverse events. The definition of reversal of infection referred to resolution of all signs, symptoms and laboratory assessment of infection or recovery from sepsis, which varied among trials due to different origins of sepsis. Adverse events were defined as organ dysfunction that was life threatening, required treatment and prolongation hospitalization, or was associated with death of the patient.

### Statistical analysis

Analyses were on an intention-to-treat basis. We calculated a weighted treatment effect across trials using fixed-effect model. The results were expressed as relative risks (RRs) with 95% confidence intervals (CIs) for dichotomous outcomes. We considered using random-effects model only in case of heterogeneity (*P-*value of χ^2 ^test less than 0.10 and I^2 ^greater than 50%). Potential sources of heterogeneity were identified by subgroup analysis on the basis of G-CSF and GM-CSF or/and by sensitivity analyses performed by omitting one study in each turn and investigating the influence of a single study on the overall meta-analysis estimate. Publication bias was assessed using funnel plots. A *P-*value of less than 0.05 was considered statistically significant. All statistical analyses were performed using Review Manager, version 5.0 (RevMan, The Cochrane Collaboration, Oxford, UK).

## Results

### Study identification

**Figure 1:**
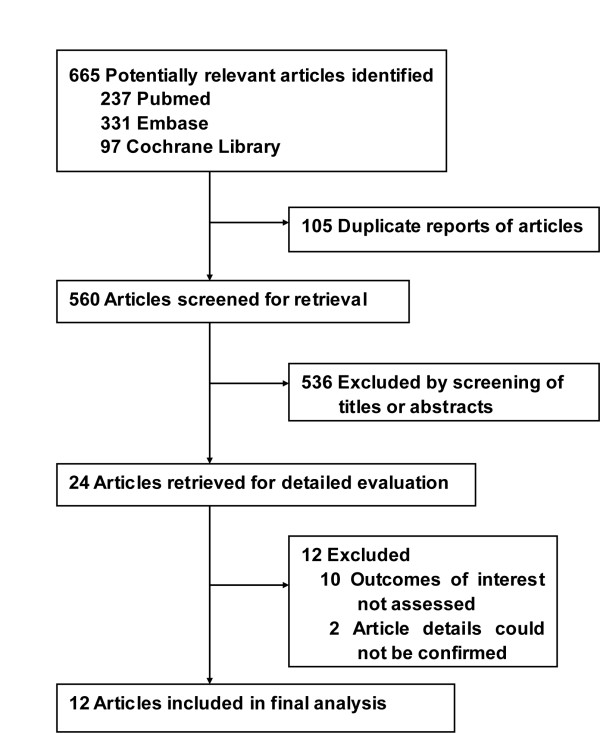
**Flow-chart of study selection**.

The comprehensive search yielded a total of 665 relevant publications, and the abstracts were obtained for all of these (Figure 1). Finally, there were 12 RCTs that met the inclusion criteria [37-48]. The Cohen К statistic for agreement on study inclusion was 0.92.

Among the selected trials, three trials were conducted in North America [40,44,45], two in Europe [43,48], two in Asia [39,46] and two in Australia [41,47]. Five trials were multicenter studies [37,38,40,42,48]. Seven trials of the included 12 trials presented the information of trial sample calculation of various clinical outcome indices based on statistical principle (14-day mortality: 1 trial [43]; 28-day mortality: 2 trials [42,46]; in-hospital mortality: 1 trial [47]; reverse rate from infection: 2 trials [37,38]; others: 1 trial [48]). Trial samples ranged widely (18 to 756 patients) with six trials enrolling fewer than 50 patients [39-41,43,44,48]. Of the 2,380 participants included, 1,188 were randomized to receive G-CSF or GM-CSF (1,113 received G-CSF and 75 received GM-CSF) and 1,192 were randomized to receive placebos. Mean age of patients ranged from 43.2 to 64.5 years. Among the included 12 trials, 8 trials were designed to compare G-CSF with placebo and 4 trials compared GM-CSF with placebo. G-CSF administration included two regimens (Lenograstim, 263 μg/day; Filgrastim, 300 μg/day) and three of four trials investigating GM-CSF versus placebo administered GM-CSF with the regimen of 3 μg/kg/day, the remaining trial 4 μg/kg/day [48]. Patients' baseline characteristics in comparative groups were well balanced, such as the Acute Physiology and Chronic Health Evaluation (APACHE) II score. Only one trial investigating GM-CSF versus placebo had discrepant baseline characteristics, in which the mean ages were 62 and 46.5 years old, respectively [41]. Details of the included studies are summarized in Table 1.

**Table 1:** Characteristics of included randomised controlled trials

				Patients			
					
Study	Design	Treatment groups	APACHE II score	N	Age (Y)	Male/female	Intervention
Nelson 1998 [37]	2 parallel groups, 71 centers	G-CSF	16.1	380	63.4 ± 16.4	235/145	Filgrastim: 300 μg/d, subcutaneous, 10 days
		Placebo	17.0	376	64.5 ± 15.2	254/122	
Nelson 2000 [38]	2 parallel groups, 105 centers	G-CSF	14.5	237	61.1 ± 17.3	138/99	Filgrastim: 300 μg/d, subcutaneous, 10 days
		Placebo	15.5	243	62.3 ± 17.4	153/90	
Tanaka 2001 [39]	2 parallel groups, 1 center	G-CSF	18.0	12	49.8 ± 6.4	11/1	Lenograstim: 2 μg/kg/d, intravenous, 5 days
		Placebo	15.9	13	54.8 ± 5.7	9/4	
Wund 2001 [40]	2 parallel groups, 3 centers	G-CSF	25.0	12	49.5	8/4	Filgrastim: 300 μg/d, intravenous, 10 days
		Placebo	31.5	6	56.5	4/2	
Presneill 2002 [41]	2 parallel groups, 1 center	GM-CSF	12	10	62	7/3	Molgramostim:3 μg/kg/d, intravenous, 5 days
		Placebo	10	8	46.5	6/2	
Root 2003 [42]	2 parallel groups, 96 centers	G-CSF	24.3 ± 7.5	348	58.9 ± 17.1	240/108	Filgrastim: 300 μg/d, intravenous, 5 days
		Placebo	24.2 ± 6.9	353	60.0 ± 16.4	247/106	
Hartma 2005 [43]	2 parallel groups, 1 center	G-CSF	13	13	66	2/11	Filgrastim: 300 μg/d, subcutaneous, 7 days
		Placebo	12.5	16	63	5/11	
Rosen 2005 [44]	2 parallel groups, 1 center	GM-CSF	_	18	56	8/10	Sargramostim: 3 μg/kg/d, intravenous, 3 days
		Placebo	_	15	52	9/6	
Orozco 2006 [45]	2 parallel groups, 1 center	GM-CSF	7.3 ± 6.3	28	43.2 ± 15.9	13/15	Molgramostim: 3 μg/kg/d, subcutaneous, 4 days
		Placebo	7.7 ± 6.4	30	49.2 ± 16.5	16/14	
Cheng 2007 [46]	2 parallel groups, 1 center	G-CSF	22	30	61	_	Lenograstim: 263 μg/d, intravenous, 3 days
		Placebo	22	30	57	_	
Stephens 2008 [47]	2 parallel groups, 1 center	G-CSF	22.5 ± 7.6	81	51.0 ± 15.1	45/36	Lenograstim: 263 μg/d, intravenous, 10 days
		Placebo	23.4 ± 8.2	83	48.9 ± 16.1	45/38	
Meisel 2009 [48]	2 parallel groups, 3 centers	GM-CSF	21.3 ± 6.1	19	64.0 ± 13.6	16/3	GM-CSF: 4 μg/kg/d, subcutaneous, 8 days
		Placebo	22.5 ± 6.6	19	63.3 ± 14.2	15/4	

**Figure 2:**
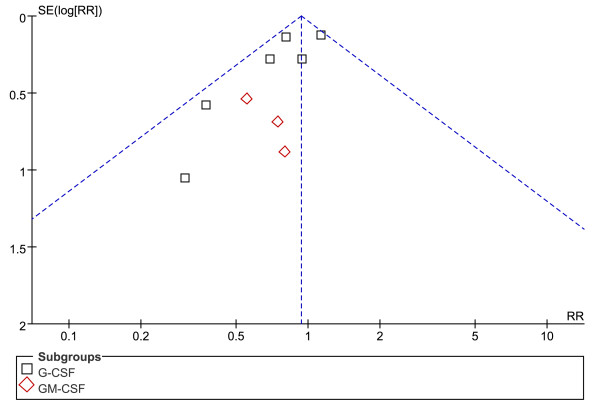
**Funnel plot of the meta-analysis**. Funnel plot for the outcome of 28-day mortality associated with G-CSF or GM-CSF therapy compared with placebo in patients with sepsis.

Randomized allocation sequence was adequately generated in six trials [41-43,46-48], for the other six trials it was judged to be unclear based on the available documents [37-40,44,45]. Allocation sequences concealment was adequately reported in six trials [41,43,44,46-48] and was judged to be unclear in the other six trials [37-40,42,45]. It was clearly stated that blinded fashion was conducted in all but one trial [44] and the outcome measurements were not likely to be influenced by lack of blinding. The numbers and reasons for withdrawal/dropout were detailed reported in all but one trial [42]. None had stopped early due to data-dependent process or other problems, so free of other sources of bias were defined across trials. Therefore, five trials [41,43,46-48] were determined as low risk of bias (plausible bias unlikely to seriously alter the results), and six trials [37-40,42,45] were at unclear risk of bias (plausible bias that rises up to some doubt about the results), while one trial [44] was at high risk of bias (plausible bias that seriously weakens confidence in the results). An overview of the quality appraisal was shown in Table 2. For the meta-analysis of G-CSF or GM-CSF therapy on 28-day mortality, there was evidence of significant funnel plot asymmetry (Figure 2).

**Table 2:** Assessing risk of bias

First author year	Sequence generation	Allocation concealment	Blinding	Incomplete outcome data addressed	Selective outcome reporting	Free of other bias	Summary risk of bias
Nelson 1998 [37]	Unclear	Unclear	Yes	Yes	Yes	Yes	Unclear
Nelson 2000 [38]	Unclear	Unclear	Yes	Yes	Yes	Yes	Unclear
Tanaka 2001 [39]	Unclear	Unclear	Unclear	Yes	Yes	Yes	Unclear
Wund 2001 [40]	Unclear	Unclear	Yes	Yes	Yes	Yes	Unclear
Presneill 2002 [41]	Yes	Yes	Yes	Yes	Yes	Yes	Low
Root 2003 [42]	Yes	Unclear	Yes	Unclear	Yes	Yes	Unclear
Hartma 2005 [43]	Yes	Yes	Yes	Yes	Yes	Yes	Low
Rosen 2005 [44]	Unclear	Yes	No	Yes	Yes	Yes	High
Orozco 2006 [45]	Unclear	Unclear	Yes	Yes	Yes	Yes	Unclear
Cheng 2007 [46]	Yes	Yes	Yes	Yes	Yes	Yes	Low
Stephens 2008 [47]	Yes	Yes	Yes	Yes	Yes	Yes	Low
Meisel 2009 [48]	Yes	Yes	Yes	Yes	Yes	Yes	Low

### All-cause mortality at 14 days

In regard to 14-day mortality, only four trials [40,43-45] consisting of a total of 138 patients evaluated the short-term outcome. The average sample size for one trial was 34 patients (sample sizes ranged from 18 to 58). Although one [43] of the four trials had calculated sample size according to 14-day mortality in the protocol, fewer patients were enrolled due to lower mortality and recruitment frequency than anticipated eventually. Hence, the meta-analysis of these four trials might be inappropriate to reveal the mortality benefit due to the limited numbers of patients. Moreover, none of the four trials reported a significant benefit in 14-day mortality following GCSF or GM-CSF administration. A total of 9 death events occurred among 71 patients (12.7%) in the treatment group compared with 13 events among 67 patients (19.4%) in the placebo group.

### All-cause mortality at 28 days

**Figure 3:**
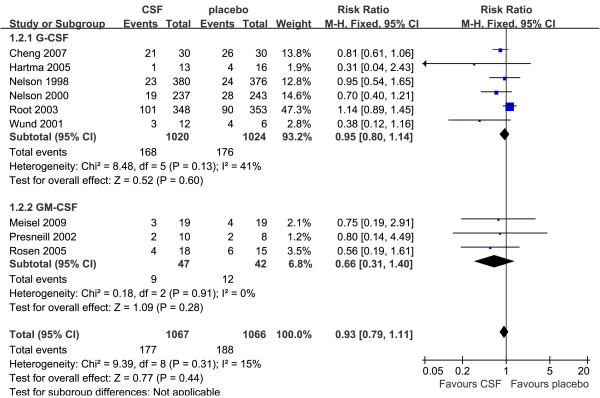
**28-day mortality of G-CSF or GM-CSF therapy versus placebo**. Fixed-effect model of risk ratio (95% confidence interval) of 28-day mortality associated with G-CSF or GM-CSF therapy compared with placebo.

Data for 28-day mortality were extracted from nine trials (*n *= 2,133) [37,38,40-44,46,48]. There were 177/1,067 (16.6%) deaths in the treatment group compared with 188/1,066 (17.6%) in the placebo group. Among these trials, there was no significant difference in 28-day mortality between the treatment group and placebo group (RR = 0.93, 95% CI: 0.79 to 1.11, *P *= 0.44; *P *for heterogeneity = 0.31, I^2 ^= 15%; Figure 3). Subgroup analysis of six trials (*n *= 2,044) [37,38,40,42,43,46] showed that G-CSF therapy was not associated with a significant reduction in 28-day mortality (RR = 0.95, 95% CI: 0.80 to 1.14, *P *= 0.60; *P *for heterogeneity = 0.13, I^2 ^= 41%). Meanwhile, subgroup analysis of the other three trials of GM-CSF (*n *= 89) [41,44,48] did not show significant difference (RR = 0.66, 95% CI: 0.31 to 1.40, *P *= 0.28; *P *for heterogeneity = 0.91, I^2 ^= 0%).

### In-hospital mortality

**Figure 4:**
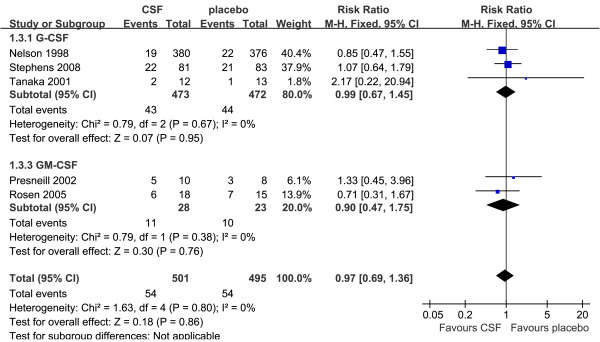
**In-hospital mortality of G-CSF or GM-CSF therapy versus placebo**. Fixed-effect model of risk ratio (95% confidence interval) of in-hospital mortality associated with G-CSF or GM-CSF therapy compared with placebo.

In-hospital mortality for patients who were treated with G-CSF or GM-CSF was 54/501 (10.8%), and that for patients treated with placebo was 54/495 (10.9%), according to five trials [37,39,41,44,47] with available data. Compared with placebo, G-CSF or GM-CSF therapy was not associated with a significant reduction in in-hospital mortality, and no heterogeneity was detected across trials (RR = 0.97, 95% CI: 0.69 to 1.36, *P *= 0.86; *P *for heterogeneity = 0.80, I^2 ^= 0%; Figure 4). Subgroup analysis of three trials (*n *= 945) [37,39,47] showed that G-CSF therapy was not associated with a significant reduction in in-hospital mortality (RR = 0.99, 95% CI: 0.67 to 1.45, *P *= 0.95; *P *for heterogeneity = 0.67, I^2 ^= 0%). Meanwhile, subgroup analysis of the other two trials of GM-CSF (*n *= 51) [41,44] did not show significant difference between the GM-CSF group and placebo group (RR = 0.90, 95% CI: 0.47 to 1.75, *P *= 0.76; *P *for heterogeneity = 0.38, I^2 ^= 0%).

### Reversal rate from infection

**Figure 5:**
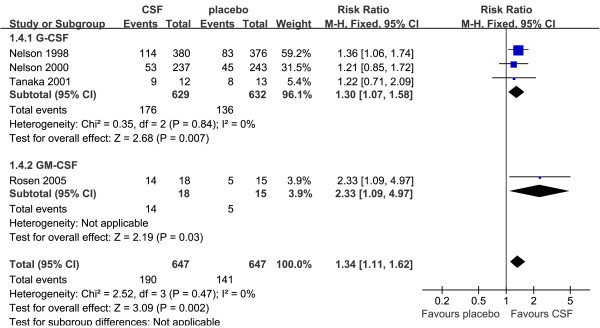
**Reversal rate from infection of G-CSF or GM-CSF therapy versus placebo**. Fixed-effect model of risk ratio (95% confidence interval) of reversal rate from infection associated with G-CSF or GM-CSF therapy compared with placebo.

Data for reversal rate from infection were available from four studies [37-39,44]. The incidence of reversal from infection in treatment group was 190/647 (29.4%) and placebo was 141/647 (21.8%). Compared with placebo, G-CSF or GM-CSF therapy was associated with a significant increase in reversal rate from infection, and no heterogeneity was detected across trials (RR = 1.34, 95% CI: 1.11 to 1.62, *P *= 0.002; *P *for heterogeneity = 0.47, I^2 ^= 0%; Figure 5). Subgroup analysis of three trials (*n *= 1,261) [37-39] showed that G-CSF therapy was associated with a significant increase in reversal rate from infection (RR = 1.30, 95% CI: 1.07 to 1.58, *P *= 0.007; *P *for heterogeneity = 0.84, I^2 ^= 0%). The other one trial of GM-CSF (*n *= 33) [44] also show significant difference (RR = 2.33, 95% CI: 1.09 to 4.97, *P *= 0.03).

### Adverse events

**Figure 6:**
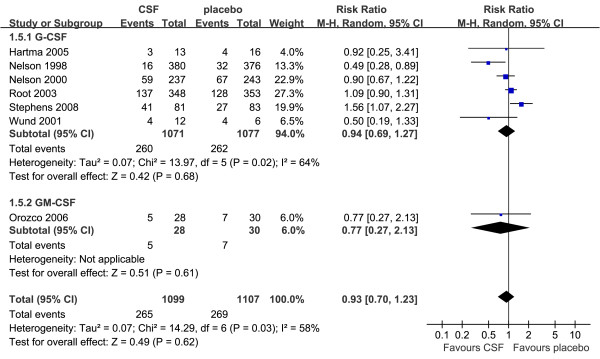
**Adverse events of G-CSF or GM-CSF therapy versus placebo**. Random-effects model of risk ratio (95% confidence interval) of adverse events associated with G-CSF or GM-CSF therapy compared with placebo.

Overall, there were no significant differences in adverse events between treatment group and placebo group according to the data from seven trials (RR = 0.93, 95% CI: 0.70 to 1.23, *P *= 0.62) [37,38,40,42,43,45,47], with heterogeneity among the trials (*P *for heterogeneity = 0.03, I^2 ^= 58%). On the basis of the results of the sensitivity analysis, one study was excluded [37]. Exclusion of the study did not significantly change the results of adverse events (RR = 1.05, 95% CI: 0.84 to 1.32, *P *= 0.44; *P *for heterogeneity = 0.17, I^2 ^= 36%; Figure 6).

## Discussion

During the past few decades, there were a variety of trials investigating the effect of G-CSF or GM-CSF therapy in patients with sepsis. However, consistent results have not been reported and no individual study has definitively established whether G-CSF or GM-CSF bring clinically important benefits to septic patients. In the present meta-analysis, we found no significant differences in all-cause mortality at 14 days or 28 days, in-hospital mortality, or adverse events between the G-CSF or GM-CSF group and placebo group in adult patients with sepsis. However, our result indicated that G-CSF or GM-CSF therapy was associated with a significant increase in reversal rate from infection.

With respect to mortality, a previous meta-analysis suggested that addition of G-CSF or GM-CSF to antibiotic therapy in preterm infants with suspected systemic infection did not significantly reduce all cause mortality at 14 days or in-hospital mortality [31]. Recently, another meta-analysis showed that administration of G-CSF was not associated with improved 28-day mortality in adults with pneumonia [49]. Our meta-analysis, which included 12 relevant RCTs comparing G-CSF or GM-CSF with placebo, demonstrated that G-CSF or GM-CSF therapy did not significantly reduce all cause mortality at 14 days or 28 days or in-hospital mortality in patients with sepsis. However, a previous trial showed that receipt of G-CSF was associated with a longer duration of survival (*P *= 0.05) in severe septic patients due to melioidosis [46]. Meanwhile, another study demonstrated that GM-CSF significantly reduced the length of in-hospital stay in patients with nontraumatic abdominal sepsis (*P *<0.001) [45]. However, it should be noted that except one trial by Meisel *et al. *[48], none of the included trials in this meta-analysis were designed with patient stratification, whereas drug efficacy should be assessed only in patients with beforehand established impairment in monocytic functions [14]. Immunological biomarkers were thus needed that allow guidance of immunotherapy, risk stratification, and determination of which individuals might benefit from a given intervention [7]. As opposed to circulating cytokines, the major advantage of measuring mHLA-DR was that its level of expression was resultant of the sum of the effects of multiple mediators during septic shock [14]. At present, there was a general consensus that a diminished mHLA-DR expression may be a reliable marker for immunosuppression in critically ill patients [50,51]. Recently, Meisel and colleagues [48] explored the GM-CSF therapy versus placebo in sepsis, the first mHLA-DR-guided immunostimulatory treatment, indicating that administration of GM-CSF to patients in the immunosuppressive phase of sepsis reversed the characteristic monocyte deactivation as demonstrated by an increase in mHLA-DR levels and Toll-like receptor-4- and Toll-like receptor-2-induced cytokine production, and reduced time of mechanical ventilation as well as length of hospital and intensive care unit stay. However, their study was not designed to explore the survival benefits of GM-CSF in sepsis so that it was insufficiently powered to evaluate mortality. Therefore, more RCTs with large number of patients to evaluate clinical parameters and mortality as primary endpoints were needed to investigate the effects of mHLA-DR-guided G-CSF or GM-CSF therapy in patients with sepsis-associated immunosuppression.

Treatment with G-CSF or GM-CSF enhanced cellular functions that were critical in infectious diseases, such as neutrophil, monocyte, and macrophage activation and increased circulating white blood cells [52-54]. In a series of nine consecutive septic patients with immunosuppression, the administration of GM-CSF induced a sustained mHLA-DR recovery, which was accompanied by a restoration in *ex vivo *tumor necrosis factor (TNF)-α production after LPS challenge [55]. Moreover, in surgical patients, Schneider *et al. *observed that G-CSF-induced restoration of mHLA-DR was not only accompanied by an increased lymphocyte proliferation and Th1 cytokine production (interleukine (IL)-2 and interferon (IFN)-γ) in response to PHA but also by a better capacity to release inflammatory cytokines in a whole blood model after LPS challenge [56]. Theoretically, G-CSF and GM-CSF might benefit patients with sepsis-associated immunosuppression and result in markedly increase in reversal rate from infection. Recently, Orozco *et al. *demonstrated that addition of GM-CSF to the standard treatment of patients with nontraumatic abdominal sepsis reduced the rate of infectious complications, shorten the duration of antibiotic therapy and the length of hospital stay [45]. Rosen and colleagues [[44] observed a higher leukocyte count, increased mHLA-DR, and better cure/improvement of infection in GM-CSF group. Results from our present meta-analysis were in line with the above results and revealed a significant increase in reversal rate from infection with G-CSF or GM-CSF therapy verse placebo (RR = 1.34, 95% CI: 1.11 to 1.62, *P *= 0.002). However, it did not bring a significant benefit in 28-day mortality (RR = 0.93, 95% CI: 0.79 to 1.11, *P *= 0.44). Due to the complexity of sepsis and associated complications, the severity of sepsis varied largely. Therefore, G-CSF or GM-CSF therapy might not be able to bring a significant mortality benefit if administrated regardless of the specific situation in individual patient, especially the immunological state of enrolled patients.

Administration of G-CSF or GM-CSF theoretically may increase the prevalence of adverse events which include immunologically mediated organ dysfunction, such as acute respiratory distress syndrome (ARDS), to which patients with severe sepsis are particularly prone [49]. However, Tanaka *et al *demonstrated that G-CSF caused leukocyte stiffness but attenuated inflammatory response without inducing lung injury in septic patients [39]. Another two studies showed that administration of G-CSF appeared to be safe in patients with pneumonia and severe sepsis [40,42]. Recently, Presneill *et al. *demonstrated that low-dose GM-CSF was associated with improved gas exchange without pulmonary neutrophil infiltration and was not associated with worsened acute respiratory distress syndrome or the multiple organ dysfunction syndromes in patients with sepsis-associated respiratory dysfunction [41]. Consistent with these studies, our meta-analysis suggested that G-CSF or GM-CSF therapy did not significantly increase the rate of adverse events (RR = 0.93, 95% CI: 0.70 to 1.23, *P *= 0.62; P for heterogeneity = 0.03, I^2 ^= 58%). Definitions of adverse events in included studies were heterogeneous and sometimes absent, which might bring about heterogeneity. Sensitivity analysis did not significantly change the results of adverse events (RR = 1.05, 95% CI: 0.84 to 1.32, *P *= 0.44; *P *for heterogeneity = 0.17, I^2 ^= 36%).

There are several limitations that need to be considered in the present study. First, the geographic regions covered in this meta-analysis included North America (United States, Mexico and Canada), Europe (Belgium, Germany and Spain), Asia (Japan and Thailand) and Oceania (Australia). Therefore, our results limited generalizability to other regions (for example, Africa and Latin America). Second, considerable heterogeneity existed in the type and dosage of G-CSF and GM-CSF. Different baseline characteristics such as age and APACHE II score might have affected the outcome of patients' response to medical management and might have produced possible clinical heterogeneity. Finally, the sample sizes were highly variable across trials and the trial enrolling the maximum number of patients [37] contained 42 times as many subjects as the two trials enrolling the minimum number of patients [40,41].

## Conclusions

While this meta-analysis demonstrated that G-CSF or GM-CSF therapy significantly increased the reversal rate from infection, it was not associated with a significant reduction in all cause mortality at 14 days or 28 days, in-hospital mortality or adverse events in patients with sepsis. Therefore, our present meta-analysis did not suggest routine use of G-CSF or GM-CSF in patients with sepsis. Large prospective multicenter clinical trials investigating mHLA-DR-guided G-CSF or GM-CSF therapy in patients with sepsis-associated immunosuppression are warranted.

## Key messages

• The literature shows that G-CSF or GM-CSF result in no differential effectiveness in treating sepsis, in terms of all-cause mortality at 14 days or 28 days, in-hospital mortality or adverse events. However, G-CSF or GM-CSF therapy significantly increased the reversal rate from infection in patients with sepsis.

• There was a lack of consensus in the literature in regard to the effect of G-CSF or GM-CSF versus placebo in treating sepsis in adults.

• Larger prospective randomized controlled trials investigating monocytic HLA-DR (mHLA-DR)-guided G-CSF or GM-CSF therapy in sepsis are warranted.

## Abbreviations

CIs: confidence intervals; G-CSF: granulocyte-colony stimulating factor; GM-CSF: granulocyte-macrophage colony stimulating factor; LPS: lipopolysaccharide; mHLA-DR: monocytic HLA-DR; PHA: phytohemagglutinin; RCTs: randomized control trials; RRs: relative risks.

## Competing interests

The authors declare that they have no competing interests.

## Authors' contributions

LB and FW conceived the study and wrote the manuscript. LB, FW and JZ designed and performed searches and participated in the extraction and analysis of the data. Both XD and JL designed the study, supervised all of the study work and statistical analysis, and helped with manuscript revisions. All authors read and approved the final manuscript.
